# Expression of Cholinergic Markers and Characterization of Splice Variants during Ontogenesis of Rat Dorsal Root Ganglia Neurons

**DOI:** 10.3390/ijms22115499

**Published:** 2021-05-23

**Authors:** Veronica Corsetti, Carla Perrone-Capano, Michael Sebastian Salazar Intriago, Elisabetta Botticelli, Giancarlo Poiana, Gabriella Augusti-Tocco, Stefano Biagioni, Ada Maria Tata

**Affiliations:** 1 Department of Biology and Biotechnology Charles Darwin, “Sapienza” University of Rome, 00185 Rome, Italy; v.corsetti@libero.it (V.C.); michaelsebastian.salazarintriago@uniroma1.it (M.S.S.I.); botticellielisabetta9@gmail.com (E.B.); giancarlo.poiana@uniroma1.it (G.P.); gabriella.tocco@fondazione.uniroma1.it (G.A.-T.); stefano.biagioni@uniroma1.it (S.B.); 2 Department of Pharmacy, University of Naples Federico II, 80138 Naples, Italy; carla.perrone@unina.it; 3 Institute of Genetics and Biophysics “Adriano Buzzati Traverso”, CNR, 80131 Naples, Italy; 4 Research Center of Neuroscience Daniel Bovet, Sapienza University of Rome, 00185 Rome, Italy

**Keywords:** cholinergic locus, sensory neurons, acetylcholine, choline acetyltransferase, vesicular acetylcholine transporter, splice variants

## Abstract

Dorsal root ganglia (DRG) neurons synthesize acetylcholine (ACh), in addition to their peptidergic nature. They also release ACh and are cholinoceptive, as they express cholinergic receptors. During gangliogenesis, ACh plays an important role in neuronal differentiation, modulating neuritic outgrowth and neurospecific gene expression. Starting from these data, we studied the expression of choline acetyltransferase (ChAT) and vesicular ACh transporter (VAChT) expression in rat DRG neurons. ChAT and VAChT genes are arranged in a “cholinergic locus”, and several splice variants have been described. Using selective primers, we characterized splice variants of these cholinergic markers, demonstrating that rat DRGs express R1, R2, M, and N variants for ChAT and V1, V2, R1, and R2 splice variants for VAChT. Moreover, by RT-PCR analysis, we observed a progressive decrease in ChAT and VAChT transcripts from the late embryonic developmental stage (E18) to postnatal P2 and P15 and in the adult DRG. Interestingly, Western blot analyses and activity assays demonstrated that ChAT levels significantly increased during DRG ontogenesis. The modulated expression of different ChAT and VAChT splice variants during development suggests a possible differential regulation of cholinergic marker expression in sensory neurons and confirms multiple roles for ACh in DRG neurons, both in the embryo stage and postnatally.

## 1. Introduction

Acetylcholine (ACh) was the first molecule identified as a neurotransmitter [1]. In recent years, it has received much attention for its presence not only in cholinergic neurons, but also in non-neuronal cells [1,2]. ACh has also been reported to be present in a large variety of animals and plants, leading to the concept of its role as a local mediator [3]. In this respect, the possible mediator role of ACh, as for other neurotransmitters, during neurogenesis has also been reported [4,5,6,7]. Studies on two experimental systems, neuroblastoma cell lines and dorsal root ganglia neurons, have shown that ACh contributes to the progression of neuron differentiation, regulating neurospecific gene expression [8,9,10,11]. ACh release, however, requires transport into a vesicular compartment, which is mediated by a specific protein, the vesicular ACh transporter (VAChT), responsible for its accumulation into synaptic vesicles. VAChT expression is thus instrumental for ACh release, as well as ACh synthesizing enzyme choline acetyltransferase (ChAT).

In cholinergic neurons, VAChT is characterized by a strict relation to the ACh synthesizing enzyme ChAT, due to the peculiarity of the two genes’ organization in the cholinergic gene locus [12,13,14]. VAChT gene localization within the first intron of ChAT, first discovered in *C. elegans*, has been reported to be a common feature of the cholinergic system (including in humans and rats) and appears to be relevant for a common regulation of the two functionally related genes [13]. The cholinergic locus shows a very complex organization, with multiple promoters and mRNA alternative splicing, leading to several ChAT and VAChT variants with identical function [12].

The question of the coupled or differential regulation of the two genes has been also discussed. Both genes are upregulated by ciliary neurotrophic factor (CNTF), retinoic acid (RA), and dibutirryl-cAMP [15], although cAMP upregulation appears more marked for VAChT [16]. On the other hand, only ChAT mRNA levels are increased by estrogen in RA-treated NG108-15 cells, while no effect on VAChT has been observed [17]. ChAT activity can be also modulated in chick DRG neurons by nerve growth factors (NGF), suggesting a possible regulated expression of the cholinergic markers, also mediated by neurotrophic factors [18].

We have previously reported the expression of ChAT and VAChT in dorsal root ganglia (DRG) [19,20], as well as the ability of developing DRG neurons to respond to ACh via muscarinic receptor activation, leading to the upregulation of neuronal markers such as neurofilament proteins [9,21,22]. Considering the complexity of the two genes’ organization and regulation, we characterized the expression of different splice variants for ChAT and VAChT during the ontogenesis of rat DRG. The analysis by RT-PCR indicated the modulated expression of different splice variants, suggesting the fine regulation of the two genes during primary sensory neuron maturation.

## 2. Results

### 2.1. Analysis of VAChT and ChAT Splice Variants

**Figure 1:**
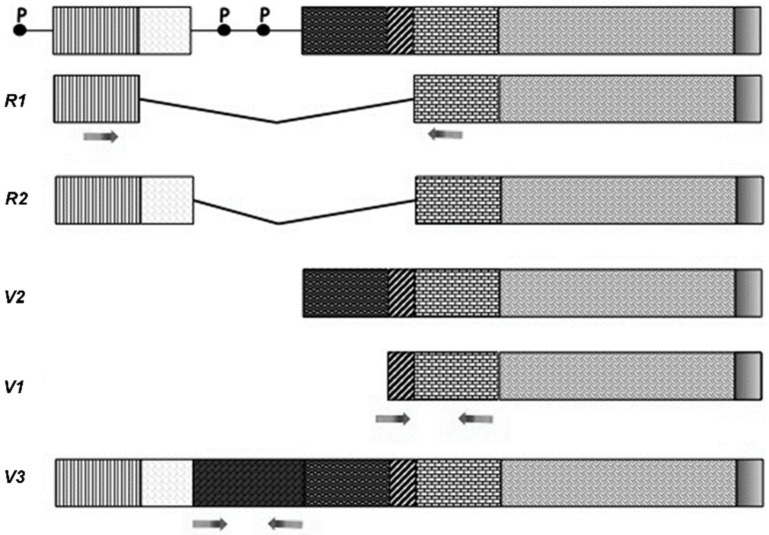
Schematic representation of the *VAChT* gene and different rat splice variants. P indicates the different putative promoter sites. The arrows indicate the sites where the primers were designed.

**Figure 2:**
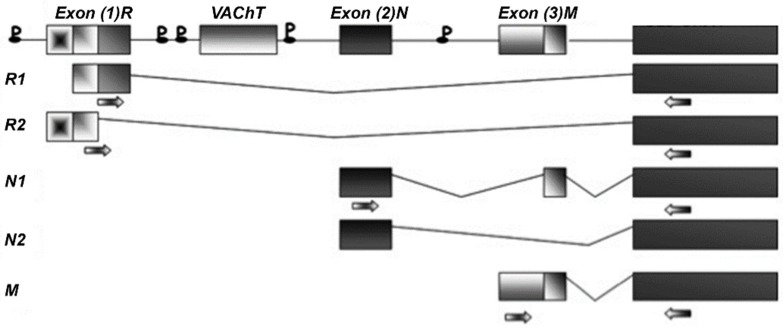
Schematic representation of the cholinergic gene locus. The *VAChT* gene is expressed in the first intron of *ChAT* gene. P indicates the various putative promoter sites regulating *ChAT* splice variant expression. Some of these promoters are in common with the VAChT gene. The arrows indicate the sites where the primers were designed.

As has been previously reported [12], five splicing forms have been described for both rat *VAChT* and *ChAT* genes. For *VAChT*, two R- forms (R1 and R2) and three V- forms (V1, V2, and V3) were identified, while for *ChAT*, R1, R2, N1, N2, and M forms were described. The *VAChT* sequences identifying each of the five forms are known, and selective primers were designed accordingly (Table S1 supplementary materials; additional information is reported in the materials and methods section). Unfortunately, the V- forms are highly homologous, so it is very difficult to discriminate them completely; therefore, a pair of primers that identified all V- forms was designed (labelled V1). Primers for V3 were designed based on a unique intronic region that is transcribed in this isoform (see Figure 1). The *ChAT* R- forms, which are less common, differ from each other by only 80 bp; therefore, only one pair of primers was designed, which generated two amplicons, one of 413 bp, (R1) and one of 493 bp (R2) (Figure 2).

**Figure 3:**
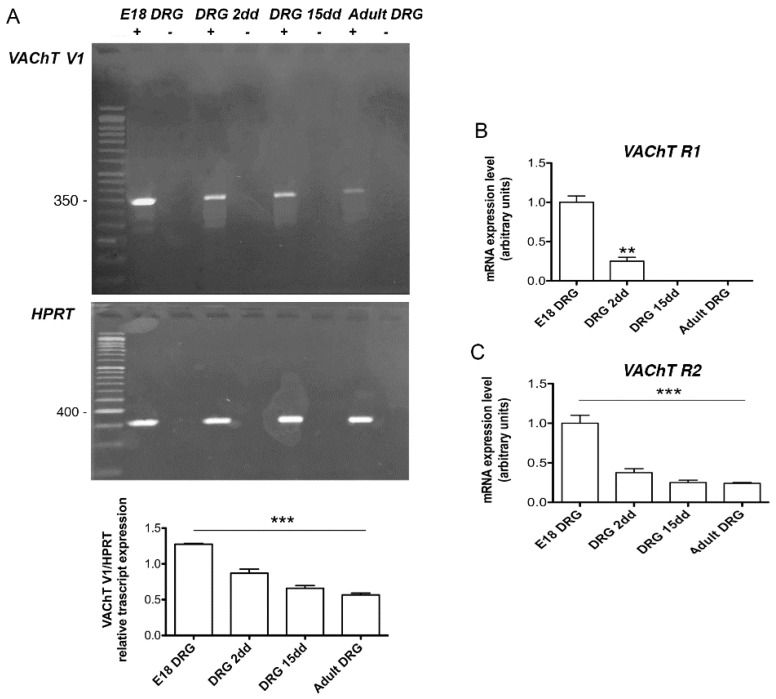
(**A**) Representative RT-PCR analysis of V1 *VAChT* splice variants at embryo E18, postnatal 2 and 15, and in adult DRG. HPRT was used as a housekeeping gene. The graph below reports the densitometric analysis of the bands normalized with respect to the HPRT housekeeping gene. The data are the mean of three independent experiments (*** *p* < 0.001); (**B**,**C**) Analysis by qRT-PCR of the R1 and R2 *VAChT* splice variants. The samples were compared with the respective E18 DRG sample. The data are the mean of three independent experiments performed in triplicate. (** *p* < 0.01; *** *p* < 0.001).

The analysis of *VAChT* expression by RT-PCR in DRG at different developmental stages (embryonic E18; postnatal 2 and 15 dd, adult 60 dd) highlighted that R- and V3 forms are apparently not expressed (data not shown), while the V1 specific transcripts are present at all examined developmental stages, the band of 352 bp is, however, more abundant in the E18 embryonic stage (Figure 3A). To exclude the presence of genomic DNA contamination, PCR analyses were performed, omitting either the reverse transcriptase or the RNA. As indicated in Figure 3A (lane-) no band was revealed, indicating the absence of genomic DNA contamination.

**Figure 4:**
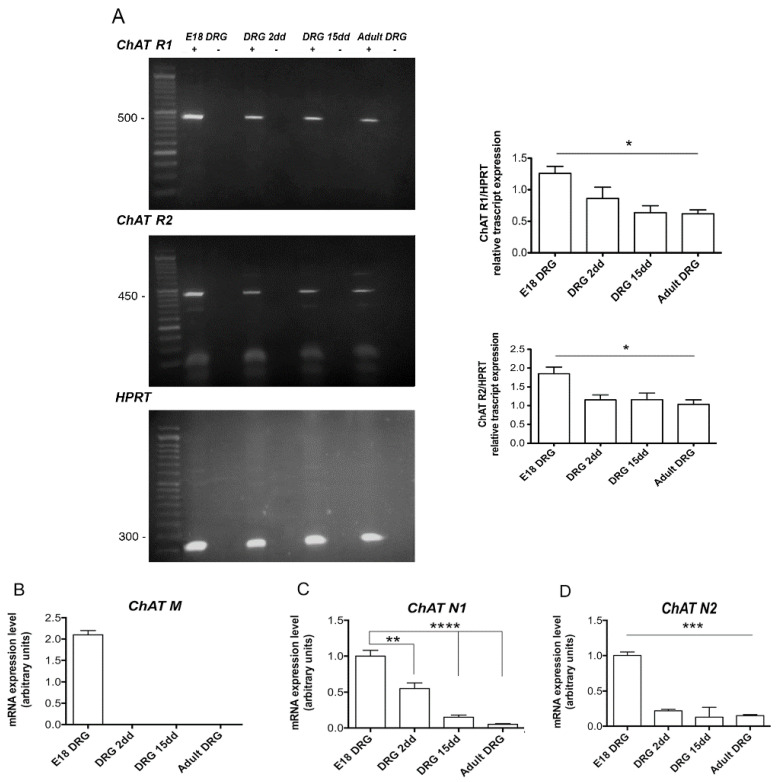
(**A**) Representative RT-PCR of the *ChAT* splice variants (R1, R2). HPRT was used as a housekeeping gene. The graphs indicate that the densitometric analysis of the bands normalized with respect to the HPRT housekeeping gene. The data are the mean of three independent experiments (* *p* < 0.001); (**B**–**D**) Analysis by qRT- PCR of the M, N1, and N2 *ChAT* splice variants, respectively. The data are the mean of three independent experiments performed in triplicate. All the samples were compared with the respective E18 DRG sample (** *p* < 0.01; *** *p* < 0.001; **** *p* < 0.0001).

However, more sensitive qRT-PCR analyses revealed that the *VAChT* R1 form was present only at E18 and 2 days postnatal, while the *VAChT* R2 form was present in all DRG developmental stages, albeit the transcript levels decreased significantly from E18 to adult (Figure 3B,C). The analysis performed for the *ChAT* gene by RT-PCR (Figure 4A) indicated that while R1 and R2 forms were expressed at higher levels at the E18 stage and progressively decreased postnatally, the M form results were absent (data not shown). However, by qRT- PCR, we observed that the M form was present only at E18 (Figure 4B). Additionally, the N1 and N2 forms were expressed in all stages, but, similarly to what observed for the *VAChT* R2 form, the level of the transcripts decreased significantly from E18 to adult (Figure 4C,D).

### 2.2. ChAT Protein Expression

**Figure 5:**
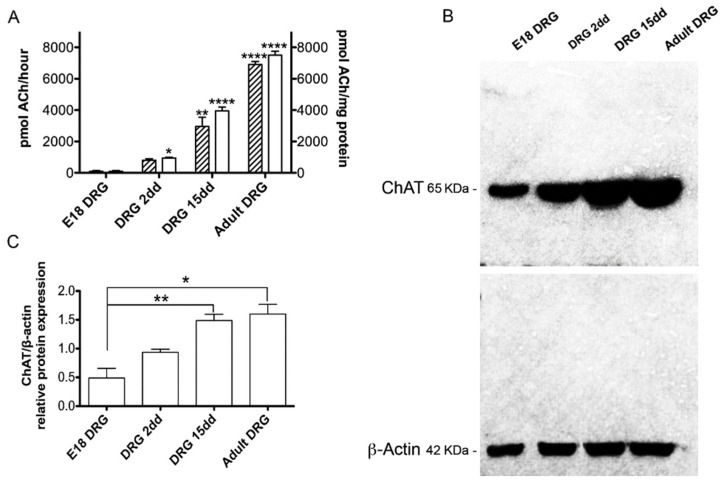
(**A**) ChAT activity was measured in DRG extracts at the E18, DRG 2dd; DRG 15dd; Adult DRGs. ChAT activity was reported as the total activity (pmol ACh tot/h) or specific activity (pmol ACh synthesized/mg of protein/h). All the samples were compared with the respective E18 DRG sample. Data are the mean ± SEM of at least 3 independent experiments performed in duplicate. (**B**) Representative Western blot analysis of the ChAT protein at E18, DRG 2dd; DRG 15dd; Adult DRGs. The graph (**C**) shows the densitometric analysis of the bands obtained after normalization with β-actin used as a protein reference. The data are the mean ± SEM of four independent experiments. All the samples were compared with the respective E18 DRG sample. (* *p* < 0.05; ** *p* < 0.01; **** *p* < 0.0001).

The expression of ChAT in rat DRG at different development stages (E18, postnatal day 2 and 15 and adult DRGs) was also evaluated by Western blot analysis. Interestingly, ChAT protein significantly increased during DRG ontogenesis (Figure 5B,C). The increase of ChAT protein in DRG was also confirmed by ChAT activity evaluation, which significantly increased from the embryonic to the adult stage, both as total activity (pmolACh tot) and as specific activity (pmolACh/mg tissue) (Figure 5A).

## 3. Discussion

Although it is well known that ACh is the neurotransmitter of cholinergic neurons, many observations indicate that it can also be synthesized by non-cholinergic neurons. DRG primary sensory neurons are peptidergic neurons; in fact, they use substance P and CGRP as their main neurotransmitters. However, our previous studies have shown that chick DRG neurons express the cholinergic markers (ChAT and VAChT) and synthesize and release ACh during development and in the adult stage [11,19,23]. Moreover, chick and rat DRGs are cholinoceptive, since they express muscarinic receptors both in neurons and in Schwann cells [20,21,24,25,26,27].

Cholinergic neurons need a coordinated expression of ChAT and VAChT to ensure that synthesized ACh is readily stored in cholinergic vesicles in order to be released when required. The discovery of a cholinergic gene locus has revealed that the synthesis of ChAT and VAChT can be finely regulated in a coordinated manner by *cis*-acting elements common to both genes [12].

On the basis of this evidence, the aim of this work was to evaluate the expression of *ChAT* and *VAChT* splicing variants during DRG development in order to establish whether the expression of the two cholinergic markers is coordinated in these neurons that do not use ACh as their main neurotransmitter.

Since the sequences of the rat splice variants for *ChAT* and *VAChT* have been described [12], we designed selective primers in order to discriminate them.

The detected *VAChT* variants V1, R1, and R2, show high levels in embryo DRGs and a marked decrease during postnatal development. The R1, R2, and N1/N2 ChAT variants follow the same pattern of expression, with high levels in embryo DRG and a drastic decrease after birth. The M variant was not detectable at any examined stage (data not shown). Although the transcription of each of these variants depends on different promoter sites and may be regulated by different factors (e.g., cAMP, RA, PKA, …) [15,16], these results suggest that the coordinated expression of these two cholinergic markers is maintained during DRG ontogenesis.

Interestingly, protein analysis showed a divergent pattern with respect to the trend of mRNA levels, at least for ChAT protein. In fact, Western blot analysis showed that, in rat DRG, ChAT protein levels constantly increased from E18 until adulthood. This pattern was confirmed by the enzyme activity assay, showing a hardly detectable level of activity at E18 and a gradually increasing activity from P2 to adult, similarly to previous observations in chick DRG [19].

As a matter of fact a further *ChAT* variant (pChAT), lacking exons 6 to 9, has been detected in DRG neurons [27,28]. Our anti-ChAT antibody was not able to discriminate this specific ChAT variant, therefore the observed variations in terms of proteins could be influenced by the expression of this additional variant.

Interestingly, the divergent pattern of expression observed, at least for ChAT transcripts and proteins, may also suggest that the post-transcriptional regulation of cholinergic markers during DRG development may occur. Although the control by miRNAs of specific neurotransmission processes remains largely unexplored, experimental evidence indicates that some miRNAs could be co-expressed and involved in regulating ACh levels [29]. To regulate cholinergic signaling and maintain ACh homeostasis, some non-coding microRNAs have been described [29,30]. Therefore, the regulation by these miRNAs may be active to finely balance the cholinergic system expression of DRG, both during development and postnatally. However, it is not possible to exclude that the decreased levels of *ChAT* and *VAChT* transcripts may be a compensatory adaptation to the increased accumulation of the respective proteins, especially in the sensory neurons.

In fact, ACh plays a relevant role in sensory neuron development and physiology; in the embryo stages, ACh may contribute to the regulation of sensory neuron maturation [4,9] and to Schwann cell proliferation and differentiation [31,32,33].

Overall, our results suggest that ACh may exert modulatory action during DRG neuron development. On the other hand, the persistent and increased expression of ChAT protein in the adult DRG is consistent with the well-known involvement of ACh in the modulation of nociceptive stimuli, as demonstrated by experiments on rat skin nerve preparation and in vitro DRG explants [34,35,36]. Moreover, the progressive increase in ChAT protein during DRG development is in agreement with the basal and potassium-evoked ACh release in synaptic and extra-synaptic regions, as previously demonstrated in chick DRG [11,23].

## 4. Materials and Methods

### 4.1. Animals

Embryos and post-natal Wistar rats were handled in accordance with the guidelines of the European Communities Council Directive (86/609/EEC of 24 November 1986) and the Italian National law DL/116/92. All methods used were carried out in accordance with the guidelines of the protocol n. 7FF2C.6.EXT.96 that has been approved by the Ministry of Health (Aut. N. 1184/2016-PR 16/12/2016). Following embryo decapitation or terminal anesthesia with CO_2_ and the cervical dislocation of the postnatal and adult rats, DRGs were dissected, collected, rapidly frozen, and stored at −80 °C for subsequent mRNA or protein analysis. Embryonal and 2 days post-natal (P2) DRGs were dissected from both males and females. Instead, P15 and adult DRGs were dissected only from male rats.

### 4.2. RNA Extraction

Total RNA was extracted from rat DRG neurons at different developmental stages by Trizol reagent (Invitrogen, Carlsbad, CA, USA) according to the manufacturer’s protocols. The RNA concentration and integrity were assessed by spectrophotometric analysis and agarose gel electrophoresis, respectively.

Total RNA was treated with the DNA-free kit (Ambion Inc., Milan, Italy) to eliminate traces of DNA contamination. Two micrograms of RNA were reverse-transcribed using random hexanucleotides as primers (Promega, Milan, Italy) in a 25 μL reaction volume containing 200 U of Moloney- murine leukemia virus reverse transcriptase (M-MLV, Promega, Milan, Italy). A negative control was included containing all reagents (including reverse transcriptase) except RNA.

### 4.3. RT-PCR and qRT- PCR Analysis

The expression of cholinergic markers of the ChAT and VAChT subtypes was evaluated by RT-PCR and qRT-PCR analyses using selective primers, as indicated in Table S1 (supplementary materials).

RNA concentration and purity were evaluated using the NanoDrop Lite Spectrophotometer (Thermo Fisher, Rome, Italy).

#### 4.3.1. RT–PCR Analysis

Reverse transcribed cDNA was amplified in a 25 µL reaction mixture containing 5× Green GoTaq buffer (Promega, Milan, Italy), 0.2 mM dNTPs (Promega, Milan, Italy), 0.4 µM of each primer, and 1 U Taq DNA polymerase (Go Taq; Promega, Milan, Italy). Each sample was amplified in duplicate. Different sets of primer pairs were used. After a first denaturing step at 95 °C for 2 min, PCR amplification was performed for different cycles organized as follows: 95 °C for 0.3 min, 56−65 °C for 0.3 min, 72 °C for 1 min, and followed by a final extension step at 72 °C for 5 min. The number of cycles was experimentally chosen in order to fall into the exponential phase of the amplification reaction. Specific pairs of primers (Table S1 supplementary materials) were used to amplify the different splicing forms; hypoxantine phosphoribosyl transferase (HPRT) gene was used as an internal standard [37]. The green labelled amplified products were separated by electrophoresis in ethidium bromide-stained 1.5% agarose gel and exposed to Chemidoc (Molecular Imager ChemiDoc XRS+ System with Image Lab Software, Biorad, CA, USA). The band intensities were quantified by optical density using ImageJ software (National Institutes of Health).

#### 4.3.2. qRT- PCR Analysis

Ten nanograms of each cDNA were used as the template for each real-time PCR reaction. SyBRGreen Jump Start Taq Ready Mix (Sigma-Aldrich, St. Louis, MO, USA) and the selective primers (final concentration 300 nM) were also added to the reaction tubes and analyzed in I Cycler IQ^TM^ Multicolor Real-Time Detection System (Bio-Rad, Hercules, CA, USA). All the samples were run in triplicate. The real-time PCR conditions consisted of a denaturing step at 95 °C for 3 min followed by 40 cycles at 95 °C for 30 s, 58 °C for 30 s, and 75 °C for 45 s. Two cycles (1 min/each) were included as final steps at 72 °C (final extension). Quantification was performed using the comparative CTmethod (CT = threshold cycle value).

#### 4.3.3. Primers

Both *ChAT* and *VAChT* genes have different splicing variants. The splicing sequences of both genes were reconstructed on the basis of published data [38,39]. Selective primers were designed in order to discriminate the R1, R2, N1, N2, and M forms of the *ChAT* gene (Figure 2) and the R, V1/V2, and V3 forms of the *VAChT* gene (Figure 1) (see also Table S1 in supplementary materials).

### 4.4. Protein Extraction and Western Blot Analysis

Total proteins were extracted by homogenizing tissue in 10 volumes of ice-cold RIPA buffer (50 mM Tris-HCl pH 8, 150 mM NaCl, 1% Triton, 2 mM EDTA, 0.1% SDS, plus a protease inhibitor cocktail (Sigma-Aldrich, St. Louis, MO, USA)) and centrifuged at 4 °C for 15 min at 1000 g. Protein concentration was determined using the Bio-Rad RC DC protein assay kit. Equal amounts of protein were subjected to a SDS-PAGE 7.5–15% linear gradient. After blotting onto a nitrocellulose membrane (Hybond-C Amersham Biosciences, Piscataway, NJ, USA), the filters were blocked in TBS containing 10% non-fat dried milk for 1 h at room temperature or overnight at 4 °C. Proteins were visualized using the appropriate primary antibody (anti-ChAT, dil 1:500 *v/v*, Immunological Sciences, Rome, Italy). The primary antibody was diluted in TBS and incubated with the nitrocellulose blot overnight at 4 °C. Then, the blot was washed and incubated for 1 h at room temperature with secondary antibodies, horseradish-peroxidase-conjugated (1:20,000 *v/v*, Promega, Madison, WI, USA). The reaction was revealed by ECL chemiluminescence reagent (Euroclone, MI, Italy). β-actin was used as a reference protein (loading control). The immunoreactive signal was revealed by exposure to photographic plate and band intensities were quantified by optical density using ImageJ software (National Institutes of Health, NIH, 469 Bethesda, MD, USA). 

### 4.5. ChAT Assay

ChAT activity was measured by a modification of the method described by Fonnum, as previously reported [18,19]. Cells were suspended in 25 mM potassium phosphate buffer (pH 7.4), lysed by freezing and thawing three times, and then centrifuged for 10 min at 20,000× *g*. Supernatants were added to the incubation mixture containing 2.5 mM choline chloride, 0.4 mM [^14^C]acetyl-CoA (56 mCi/mmol), 0.2 mM eserine sulfate, 0.3 M NaCl, and 20 mM EDTA in 0.1 M sodium phosphate buffer, pH 7.4. After incubation at 37 °C for 60 min, the reaction mixture was transferred into scintillation vials containing 10 mL of toluene, 5 g/L diphenyloxazole, 0.1 g/L 1,3-bis(4-methyl-5-phenyloxazole-2-yl) benzene, 500 mL of heptanone, 500 mg of naphthalene, and 15 mg of sodium tetraphenyl boron. The amount of [^14^C] ACh was determined using a Packard liquid scintillation counter. Protein concentration was determined using the Bio-Rad RC DC protein assay kit. Blank values were obtained either by omitting the homogenate or by incubating the protein extracts in the presence of 50 mM 4-naphthylvinyl pyridine, a specific inhibitor of ChAT activity.

### 4.6. Statistical Analysis

Data were expressed as means ± SEM and were representative of at least three independent experiments (including at least 3 or 4 animals for each experimental condition). Statistically significant differences were calculated by an unpaired independent Student’s t test, or ANOVA test followed by Bonferroni multiple comparison posttest, with *p* < 0.05 accepted as statistically significant (* *p* < 0.05; ** *p* < 0.01; *** *p* < 0.001). All statistical analyses were performed using GraphPad Prism 6 software.

## 5. Conclusions

In conclusion, the data reported here show the ability of rat DRG neurons to synthesize and store ACh in cholinergic vesicles. Although the proteins obtained from different splice variants were identical, the coordinated expression of the different splice variants for *ChAT* and *VAChT* suggests that different regulating factors (e.g., cAMP; NGF) may contribute in DRG to the modulation of the cholinergic locus expression. The increasing expression and activity of ChAT proteins during DRG ontogenesis also confirms the possible dual role of ACh; at the embryo stage and in the post-natal period, it may control neuron and glial cell differentiation and myelination [4,31,32,33], while in the adult stage, it may act as a modulator of sensory neuron neurotransmission [34,35,36].

## Supplementary Materials

The following are available online at https://www.mdpi.com/article/10.3390/ijms22115499/s1.

## Abbreviations

Ach	Acetylcholine
cAMP	Cyclic adenosine monophosphate
ChAT	Choline acetyltransferase
CGRP	Calcitonin gene related peptide
CNTF	Ciliary neurotrophic factor
DRG	Dorsal root ganglion
FBS	Fetal bovine serum
GAPDH	Glyceraldehyde phosphodehydrogenase
HPRT	Hypoxanthine phosphoribosyl transferase
NG	Neuroblastoma-glioma
NGF	Nerve growth factor
RA	Retinoic acid
SDS	Sodium dodecyl sulphate
VAChT	Vesicular acetylcholine transporter

## References

[1] Eglen R.M. (2005). Muscarinic receptor subtypes in neuronal and non-neuronal cholinergic function. Auton. Autocoid Pharmacol..

[2] Wessler I., Kirkpatrick C.J. (2008). Acetylcholine beyond neurons: The non-neuronal cholinergic system in humans. Br. J. Pharmacol..

[3] Horiuchi Y., Kimura R., Kato N., Fujii T., Seki M., Endo T., Kato T., Kawashima K. (2003). Evolutional study on acetylcholine expression. Life Sci..

[4] Biagioni S., Tata A.M., De Jaco A., Augusti-Tocco G. (2000). Acetylcholine synthesis and neuron differentiation. Int. J. Dev. Biol..

[5] Abreu-Villaça Y., Filgueiras C.C., Manhães A.C. (2011). Developmental aspects of the cholinergic system. Behav. Brain Res..

[6] Asrican B., Paez-Gonzalez P., Erb J., Kuo C.T. (2016). Cholinergic circuits control of postnatal neurogenesis. Neurogenesis.

[7] Paez-Gonzalez P., Asrican B., Rodriguez E., Kuo C.T. (2014). Identification of distinct ChAT^+^ neurons and activity dependent control of postnatal SVZ neurogenesis. Nat. Neurosci..

[8] Tata A.M., De Stefano M.E., Srubek Tomassy G., Vilarò M.T., Levey A.I., Biagioni S. (2004). Subpopulations of rat dorsal root ganglion neurons express active vesicular acetylcholine transporter. J. Neurosci. Res..

[9] Tata A.M., Cursi S., Biagioni S., Augusti-Tocco G. (2003). Cholinergic modulation of neurite outgrowth and neurofilament expression in developing chick sensory neurons. J. Neurosci. Res..

[10] Salani M., Anelli T., Augusti-Tocco G., Lucarini E., Mozzetta C., Poiana G., Tata A.M., Biagioni S. (2009). Acetylcholine-induced neuronal differentiation: Muscarinic receptor activation regulates EGR-1 and REST expression in neuroblastoma cells. J. Neurochem..

[11] Corsetti V., Mozzetta C., Biagioni S., Augusti-Tocco G., Tata A.M. (2012). Acetylcholine release: The mechanisms and the site of release during chick dorsal root ganglia ontogenesis. Life Sci..

[12] Eiden L.E. (1998). The cholinergic gene locus. J. Neurochem..

[13] Mathews E.A., Mullen G.P., Manjarrez J.R., Rand J.B. (2015). Unusual Regulation of Splicing of the Cholinergic Locus in Caenorhabditis elegans. Genetics.

[14] Candiani S., Lacalli T.C., Parodi M., Oliveri D., Pestarino M. (2008). The cholinergic gene locus in Amphioxus: Molecular characterization and developmental expression patterns. Dev. Dyn..

[15] Berse B., Blusztajn J.K. (1995). Coordinated up-regulation of choline acetyltransferase and vesicular acetylcholine transporter gene expression by the retinoic acid receptor alpha, cAMP, and leukemia inhibitory factor/ciliary neurotrophic factor signaling pathways in a murine septal cell line. J. Biol. Chem..

[16] Castell X., Cheviron N., Barnier J.V., Diebler M.F. (2003). Exploring the regulation of the expression of VAChT and ChAT genes in NG108-15 cells: Implication of PKA and PI3K signalling pathways. Neurochem. Res..

[17] Yamamuro Y., Aizawa S. (2010). Asymmetric regulation by estrogen at the cholinergic locus in differentiated NG108-15 neuronal cells. Life Sci..

[18] Biagioni S., Tata A.M., Agrati C., Cianfarani F., Augusti-Tocco G. (2000). Nerve growth factor modulation of cholinergic marker expression in dorsal root ganglia. J. Neurosci. Res..

[19] Tata A.M., Plateroti M., Cibati M., Biagioni S., Augusti-Tocco G. (1994). Cholinergic markers are expressed during development of chick dorsal root ganglia. J. Neurosci. Res..

[20] Tata A.M., Tripiciano A., Filippini A., Biagioni S., Augusti-Tocco G. (2000). Muscarinic receptors modulate intracellular calcium level in chick sensory neurons. Brain Res..

[21] Tata A.M., Vilaró M.T., Mengod G. (2000). Muscarinic receptor subtypes expression in rat and chick dorsal root ganglia. Brain Res. Mol. Brain Res..

[22] Biagioni S., Tata A.M., Augusti-Tocco G. (1999). Expression of cholinergic system components in dorsal root ganglion (DRG) neurons: Its possible dual role in development and nociception. Recent Res. Devel. Neurochem..

[23] Bernardini N., Srubek Tomassy G., Tata A.M., Augusti-Tocco G., Biagioni S. (2004). Detection of basal and potassium-evoked acetylcholine release from embryonic DRG explants. J. Neurochem..

[24] Bernardini N., De Stefano M.E., Tata A.M., Biagioni S., Augusti-Tocco G. (1998). Neuronal and non-neuronal cell populations of the avian dorsal root ganglia express muscarinic acetylcholine receptors. Int. J. Dev. Neurosci..

[25] Tata A.M., Vilaró M.T., Agrati C., Biagioni S., Mengod G., Augusti-Tocco G. (1999). Expression of muscarinic M2 receptor mRNA in dorsal root ganglia of neonatal rat. Brain Res..

[26] Loreti S., Vilaró M.T., Visentin S., Rees H., Levey A.I., Tata A.M. (2006). Rat Schwann cells express M1–M4 muscarinic receptor subtypes. J. Neurosci. Res..

[27] Bellier J.P., Kimura H. (2007). Acetylcholine synthesis by choline acetyltransferase of peripheral type as demonstrated in adult rat dorsal root ganglion. J. Neurochem..

[28] Elnasharty M.A., Sayed-Ahmed A. (2014). Expression and localization of pChAT as a novel method to study cholinergic innervation of rat adrenal gland. Acta Histochem..

[29] Nadorp B., Soreq H. (2014). Predicted overlapping microRNA regulators of acetylcholine packaging and degradation in neuroinflammation-related disorders. Front. Mol. Neurosci..

[30] Greenberg D.S., Soreq H. (2014). MicroRNA therapeutics in neurological disease. Curr. Pharm. Des..

[31] Loreti S., Ricordy R., De Stefano M.E., Augusti-Tocco G., Tata A.M. (2007). Acetylcholine inhibits cell cycle progression in cultured rat Schwann cells by activation of M2 receptor subtype. Neuron Glia Biol..

[32] Uggenti C., De Stefano M.E., Costantino M., Loreti S., Pisano A., Avallone B., Talora C., Magnaghi V., Tata A.M. (2014). M2 muscarinic receptor activation addresses Schwann cell differentiation and myelin organization. Dev. Neurobiol..

[33] Magnaghi V., Procacci P., Tata A.M. (2009). Novel pharmacological approaches to Schwann cells as neuroprotective agents for peripheral nerve regeneration. Int. Rev. Neurobiol..

[34] De Angelis F., Marinelli S., Fioretti B., Catacuzzeno L., Franciolini F., Pavone F., Tata A.M. (2014). M2 receptors exert analgesic action on DRG sensory neurons by negatively modulating VR1 activity. J. Cell Physiol..

[35] De Angelis F., Tata A.M. (2016). Analgesic effects mediated by muscarinic receptors: Mechanisms and pharmacological approaches. Cent. Nerv. Syst. Agents Med. Chem..

[36] Bernardini N., Roza C., Sauer S.K., Gomeza J., Wess J., Reeh P.W. (2002). Muscarinic M2 receptors on peripheral nerve endings: A molecular target of antinociception. J. Neurosci..

[37] Volpicelli F., Perrone-Capano C., Da Pozzo P., Colucci-D’Amato L., di Porzio U. (2004). Modulation of Nurr-1 gene expression in mesenchephalic dopaminergic neurones. J. Neurochem..

[38] Cervini R., Houhou L., Pradat P.F., Béjanin S., Mallet J., Berrard S. (1995). Specific vesicular acetylcholine transporter promoters lie within the first intron of the rat choline acetyltransferase gene. J. Biol. Chem..

[39] Kengaku M., Misawa H., Deguchi T. (1993). Multiple mRNA species of choline acetyltransferase from rat spinal cord. Brain Res. Mol. Brain Res..

