# Supplementary material for: Expression of Cholinergic Markers and Characterization of Splice Variants during Ontogenesis of Rat Dorsal Root Ganglia Neurons
Source: Int J Mol Sci. 2021 May 23;22(11):5499. doi: 10.3390/ijms22115499 (PMC8197147; doi:10.3390/ijms22115499)
Supplement: Supplementary file 1 [file ijms-22-05499-s001.zip › ijms-1242772-supplementary.pdf]

Table S1. Forward and reverse primer sequences, lenght of amplified product (bp) and the annealing temperature (Ta °C) used in RT-PCR experiments for the following genes: hypoxantine-phosphoribosyl-transferase (HPRT); the isoforms of choline acetyltransferase (R1, R2, M and N1/N2 ChAT); the isoforms of vesicular acetylcholine transporter (R1, V1 and V3 VAChT).

Gene	*Forward 5'-3'*	*Reverse 5'-3'*	*Lenght (bp)*	*Ta °C*
HPRT	CCTGCTGGATTACATTAAAGCACTG	CTTCGTGGGGTCCTTTTCACCAGC	*282 bp*	*60°C*
R1 ChAT	AGCCTTCCTAAGCCTCTACTG	CTAAGCACACCAGAGATGAGG	*521 bp*	*58°C*
R2 ChAT	GAAGGAAGGTCTACAGCTCTG	CTAAGCACACCAGAGATGAGG	*453 bp*	*58°C*
M ChAT	GAAGGCAGAAGATGGGAATCG	CTAAGCACACCAGAGATGAGG	*802 bp*	*60°C*
N1/N2 ChAT	GAGGTAGTCCCGTCTTTTAGG	CTAAGCACACCAGAGATGAGG	*767-575 bp*	*56°C*
R1 VAChT	AGCCTTCCTAAGCCTCTACTG	CTTTCCCTAAGATGCCTCCAC	*413 bp*	*58°C*
V1 VAChT	GTGGAGGAAGAGGCAAG	GCAGAGGCTAGGATAAGG	*352 bp*	*56°C*
V3 VAChT	GAGGAGTTAGATTGAGGAGTG	ATGAGGGTATGAGGACTGAG	*189 bp*	*56°C*
